# Proceedings of the Central States Dental Society

**Published:** 1868-02

**Authors:** J. F. Canine


### PROCEEDINGS OF THE CENTRAL STATES DENTAL SOCIETY.

Pursuant to call, the Central States Dental Society met in annual session at the office of Drs. Goddard and Peabody, corner of Fourth and Green streets, Louisville, Ky., at 2 o'clock Thursday, P. M. Dec. 26, 1867.

Dr. Redman in the chair, and Dr. Shadoan as Secretary.

On calling the roll, twenty-six members were found to be present.

The Executive Committee and the Secretary submitted their annual reports, which were adopted.

On motion the Code of Dental Ethics adopted by the American Dental Association, and offered as an amendment to the constitution, was taken up, discussed, and adopted.

On motion of Dr. Goddard, it was resolved that the Society meet from 9 to 1 o'clock, and from 2 to 5 o'clock, and at 7½ each day.

There being only one of the members of the Committee on Membership present, viz.: Dr. Goddard, the chairman, filled the vacancies by appointing Drs. Cobb and Russell.

The Committee reported as worthy of membership the following named Dentists, and they were duly elected:

Dr. A. T. Keightly, Greencastle, Indiana.

Dr. S. C. Taylor, Toledo, Ohio.

Dr. Rosson, Troy, Ohio.

Dr. Isaiah Forbes, St. Louis, Missouri.

Dr. C. D. Hillman, Louisville

The first of the subjects proposed for discussion, "Filling Teeth — what are the points to be observed to secure success?" was taken up for consideration. Drs. Taft, McClellan, Morgan, and others debated the question at some length, when the Society adjourned to meet at the office of Dr. Canine, Fifth street, between Green and Walnut, at 9 o'clock A. M., Friday.

SECOND DAY.

The Society met as per adjournment. Minutes of the previous day's meeting were read and approved.

On motion, the Society went into the selection of a place for holding the next semi-annual session on the second Tuesday in April next. Nashville, Tenn., Lexington, Ky., and Indianapolis, Ind., were nominated, and on the first ballot Indianapolis was chosen.

Then, according to resolution of the preceding day, officers of the Society for the ensuing year were elected, viz:

President — Dr. McClellan.

Vice Presidents — Dr. Keightly, 1st; Dr. Cobb, 2d; Dr. Naghel, 3d.

Secretary — Dr. Canine.

Corresponding Secretary — Dr. Rosson.

Treasurer — Dr. R. H. Wilson.

Drs. Goddard and Arrington were appointed to conduct the President elect to the chair. Appropriate speeches were made by both the retiring officer, Dr. Redman, and the incoming officer, Dr. McClellan.

On motion of Dr. Goddard, a vote of thanks was tendered the retiring officers.

By request, Dr. Forbes, of St. Louis, who has recently been across the waters, gave some descriptions of Dentistry in Europe, which were listened to with much interest.

Drs. Goddard, Russell and Arrington were appointed a Committee on Code of Ethics.

The subject of "Filling Teeth," under discussion the previous evening, was resumed by Drs. Wilson, Morgan, Taft, Russell, Peabody, and others. After a protracted discussion, the Society adjourned until 2 P. M.

AFTERNOON SESSION.

Having assembled according to adjournment, the above subject was renewed and further discussed by Drs. Peabody, Russell, Forbes, Arrington, and others, when on motion of Dr. Goddard the subject was dismissed, and a most valuable and interesting paper upon Microscopy, by Dr. Cutler, was read by Dr. Taft. A resolution of thanks to Dr. Cutler was adopted.

The subject of Microscopy was passed, and the second subject, Sensitive Dentine, was taken up, and debated for some time.

On motion of Dr. Redman, a committee of three to report upon new appliances was appointed, viz: Drs. S. J. Cobb, R. C. Morgan and J. H. Taylor.

The following standing committees were also appointed:

Committee on Membership — Drs. Driggs, Cobb and Peabody.

Executive Committee — Drs. Arrington, Morrell and Redman.

The Society thereupon adjourned to meet again at Dr. Wilson's office at 9 o'clock A. M., to-morrow.

THIRD DAY — FORENOON SESSION.

The Association met according to adjournment. Minutes read and approved.

Reports of Committees were called for, when the committee on membership presented the following:

Your committee would respectfully report that they have examined into the charges preferred against Dr. B. M. Gildea, and find the charges of unprofessional conduct sustained.

[Signed] S. DRIGGS, F. PEABODDY, S. J. COBB, } Committee.

The question was asked, whether Dr. Gildea had been summoned to appear for trial, when one of the committee answered that he had.

On motion, a committee was appointed to ask Dr. G. to appear, which he did.

After all being said for and against the defendant, a vote was taken which resulted in his expulsion.

On motion, the Secretary was instructed to notify other societies of the action of this association in the above case.

A motion was made to change the time of holding the annual meeting of this Society (a notice having been given one year previous) which resulted in changing the time from the 26th of December to the third Tuesday in January of each year.

Your committee would report the following names as delegates to the American Dental Association, to be held at Niagara Falls in July next: Drs. C. E. Dunn, G. S. Jones, G. F. Lampkin, E. Q Naghel, Alex. Hartman, C. P. Baird, E. W. Mason, S. C. Chisholm, J. M. Comegies, J. F. Canine.

[Signed] W. T. ARRINGTON, W. G REDMAN, F. PEABODY, } Committee.

Dr. McClellan then presented for inspection his new base for artificial teeth, which he gave some explanation in regard to durability, adaptability, &c., which was referred to the committee on new inventions and appliances.

REPORT OF THE COMMITTEE ON APPLIANCES.

Your committee, would report that we have examined into the merits of several improvements. First, an automatic mallet, presented by S. C. Taylor, of Toledo, Ohio, and find it to be a very ingenious instrument, and would recommend a trial by those using such instruments. Also, several specimens of a new base for artificial teeth, presented by Dr. J. A. McClelland, of this city, and think that it is destined to take the place of rubber, from what we can see and learn of it, and find it far superior to anything of its kind. We find it beautiful, light and strong.

On motion, the Secretary was ordered to serve notice on Dr. Abner Wessen, of Memphis, Tenn., and furnish him with a copy of the charges preferred against him in this Society.

A vote of thanks was tendered Drs. Goddard, Peabody, Canine and Wilson, for the use of their offices during the sittings of this Society.

Dr. Peabody gave notice of an additional by-law.

On motion, the Secretary was requested to furnish a copy of these proceedings to the city papers and to the Dental journals for publication.

On motion, adjourned to meet in the city of Indianapolis, Ind., on the second Tuesday in April 1868, at 10 o'clock, A. M.

J. F. CANINE, *Secretary.*

